# Conserved Histidine Adjacent to the Proximal Cluster Tunes the Anaerobic Reductive Activation of *Escherichia coli* Membrane‐Bound [NiFe] Hydrogenase‐1

**DOI:** 10.1002/celc.201800047

**Published:** 2018-02-16

**Authors:** Lindsey A. Flanagan, Harriet S. Chidwick, Julia Walton, James W. B. Moir, Alison Parkin

**Affiliations:** ^1^ Department of Chemistry University of York Heslington, York; ^2^ Department of Biology University of York Heslington, York

**Keywords:** bioinorganic chemistry, electrochemistry, electron transfer, hydrogen, metalloenzymes

## Abstract

[NiFe] hydrogenases are electrocatalysts that oxidize H_2_ at a rapid rate without the need for precious metals. All membrane‐bound [NiFe] hydrogenases (MBH) possess a histidine residue that points to the electron‐transfer iron sulfur cluster closest (“proximal”) to the [NiFe] H_2_‐binding active site. Replacement of this amino acid with alanine induces O_2_ sensitivity, and this has been attributed to the role of the histidine in enabling the reversible O_2_‐induced over‐oxidation of the [Fe_4_S_3_Cys_2_] proximal cluster possessed by all O_2_‐tolerant MBH. We have created an *Escherichia coli* Hyd‐1 His‐to‐Ala variant and report O_2_‐free electrochemical measurements at high potential that indicate the histidine‐mediated [Fe_4_S_3_Cys_2_] cluster‐opening/closing mechanism also underpins anaerobic reactivation. We validate these experiments by comparing them to the impact of an analogous His‐to‐Ala replacement in *Escherichia coli* Hyd‐2, a [NiFe]‐MBH that contains a [Fe_4_S_4_] center.

Membrane‐bound [NiFe] hydrogenases (MBH) are a family of enzymes which are found in the periplasm of bacteria where they catalyze H_2_ oxidation (H_2_ → 2H^+^+2e^−^).^1^ These H_2_‐enzymes are particularly amenable to study by protein film electrochemistry (PFE) because the rapid rate of electrocatalytic turnover acts as a highly effective signal amplifier, meaning that substantial levels of catalytic current can be detected even when only a small number of molecules have adsorbed to the electrode surface in an electroactive configuration (typical electroactive coverages are in the region of 7 pmol cm^−2^).^2^ When coupled with structural and molecular biology mutation studies, this electrochemical technique becomes a powerful assay method that can help pinpoint the role of certain amino acids in tuning the chemistry of a hydrogenase.2a In particular, the energetic efficiency of catalysis, often quantified as the “overpotential” voltage difference between the onset of catalysis and the experimental hydrogen reduction potential, *E*(H^+^/H_2_), and the catalytic “bias” (ratio of H_2_‐oxidation to H^+^‐reduction current) are readily quantified in PFE.

It is interesting to compare the two H_2_‐uptake MBH from *E. coli*, identified as Hyd‐1 and Hyd‐2, because despite high levels of structural and protein sequence similarity these enzymes have strikingly different reactivities with O_2_ (Hyd‐1 can sustain H_2_ oxidation in the presence of O_2_, Hyd‐2 cannot) and very different catalytic fingerprints (at pH>6 Hyd‐1 is a unidirectional oxidation‐only catalyst with an overpotential while Hyd‐2 is a bidirectional H_2_‐catalyst with no overpotential).^3^ Generally, identifying the relatively rare conserved points of difference between such O_2_‐tolerant and O_2_‐sensitive hydrogenases is a good starting point for understanding how nature has evolved the structures of these proteins to achieve distinct functions (Hyd‐2 can provide *E. coli* with a greater membrane potential, but Hyd‐1 is functional under a wider range of growth conditions).^3^ Ultimately, the biotechnological aim of such studies is to determine whether it is possible to program photosynthetic bacteria to produce O_2_‐functioning, H_2_‐producing enzymes, or to design such enzymes for use in biotechnological devices.^1^

The electron‐transfer iron‐sulfur cluster closest to the NiFe active site, the “proximal” cluster, has been a center of focus in mutation studies. In O_2_‐tolerant MBH such as *E. coli* Hyd‐1, this is a uniquely structured [Fe_4_S_3_Cys_2_] cluster which can reversibly access three different oxidation states (+5, +4, +3).^4^ Our previous work on *Salmonella* Hyd‐54c and work by Frielingsdorf et al on *Ralstonia* MBH^5^ showed that a histidine (His) residue in the large subunit which points directly at the proximal cluster in the small subunit of these O_2_‐tolerant enzymes (HyaB‐His229 in *E. coli* Hyd‐1, Figure 1) plays a role in allowing the enzymes to catalyze H_2_ oxidation in the presence of O_2_. In the *Ralstonia* work, a high‐resolution crystal structure of the proximal cluster in the “over‐oxidized” +5 state suggested that this is because the His residue stabilizes an OH^−^ ligand which binds to one of the Fe in the “open” structure (Figure 1).

**Figure 1:**
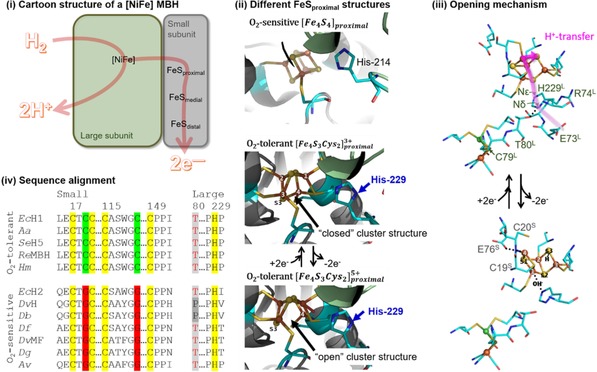
Structural similarities and differences between O_2_‐tolerant MBH and O_2_‐sensitive [NiFe] hydrogenases. i) Cartoon of the heterodimeric minimal functional unit. ii) The difference between the proximal iron sulfur cluster (FeS_proximal_) structure of O_2_‐sensitive enzymes which contain an [Fe_4_S_4_] center, and the proximal [Fe_4_S_3_Cys_2_] center of O_2_‐tolerant MBH, which can reversibly interconvert between a “closed”, +3, reduced state and an “open”, +5, “over‐oxidized” state. The relative position of His amino acids to these centers is highlighted, with *E. coli* Hyd‐2 numbering used in the O_2_‐sensitive structure which is derived from PDB 3MYR^[7]^, and *E. coli* Hyd‐1 numbering used in the O_2_‐tolerant structures which are derived from PDB 3RGW^4^ for the +5 state and PDB 4IUC^5^ for the +3 state. Colors: gray ribbon, small subunit; green ribbon, large subunit; yellow spheres and sticks, sulfur; orange spheres, iron; light blue, carbon; dark blue, nitrogen; red, oxygen. iii) Dance's proposed^6^ proton‐transfer pathway from Glu‐73 to S3 that is mediated by His‐229, which is sensitive to the active site geometry via its H‐bond to Thr‐80. Top structure based on *E. coli* Hyd‐1 PDB3UQY, and bottom on *E. coli* Hyd‐1 3USC. Green labels indicate large subunit (HyaB) residues, gray labels indicate small subunit (HyaA) residues and the location of the OH^−^ group observed by Frielingsdorf et al.^5^ is indicated. Green spheres are used to indicate nickel, all other color Scheme details are as in (ii). iv) Sequence alignments comparing the ligation of the proximal cluster of O_2_‐tolerant MBH and O_2_‐sensitive [NiFe] hydrogenase. *E. coli* Hyd‐1 numbering is used.

On the basis of these results, Dance carried out DFT calculations to probe how O_2_ binding at the NiFe active site of an O_2_ tolerant MBH induces the proximal cluster to change from the reduced +3 state to the superoxidized +5 state.^6^ He concluded that this triggering mechanism is in part mediated by proton transfer from the His to a sulfur atom of the iron‐sulfur cluster, via the nearby Cys‐19 residue that replaces an inorganic sulfur in the cluster (Figure 1).^6^ This proton transfer is calculated to induce the breaking of a S‐Fe bond within the cluster and other major structural changes such that the redox potential of the new geometry is sufficiently low that electrons can be transferred to the active site and contribute to the reduction of the inhibitory O_2_ bridging the Ni and Fe.^6^ It is concluded that via its H‐bond from Nδ to threonine‐80 (T80^L^), geometrical changes at the active site are sensed by the His residue. Proton transfer is then initiated from Glu73 (Figure 1), since the carboxylate side‐chain can relay a proton from a reservoir formed by Arg‐73 and two water molecules to or from the Nδ of His‐229.^6^

However, in our work on the *Salmonella* Hyd‐5 His‐to‐Ala variant, we noted that the extent by which the enzyme oxidatively‐inactivated, attributed to formation of the active site Ni‐B state, appeared to be enhanced regardless of whether O_2_ was present or not.4c Thus, the amino acid exchange somehow impacts on the mechanism of anaerobic formation/reactivation of the Ni‐B state (Scheme 1). We postulated that this could be related to the fact that proximal cluster over‐oxidation underpins Ni‐B state formation/activation in both oxygenic and non‐oxygenic conditions since EPR studies on other O_2_‐tolerant [NiFe]‐MBH have shown that in the absence of O_2_ the “superoxidized”, +5, open state of the proximal cluster of O_2_‐tolerant MBH is still accessible.^8^ We have therefore created an *E. coli* Hyd‐1 H229A variant to quantify the impact of this amino acid exchange on the formation and re‐activation of the inactivated Ni^3+^ Ni‐B state under O_2_‐free conditions. Alanine (Ala) is a common choice as a replacement residue when probing the biochemical function of the amino acids in a protein structure because it possesses sufficient steric bulk to stabilize the resultant variant structure but does not introduce any new acid/base or redox reactivity.

**Scheme 1:**
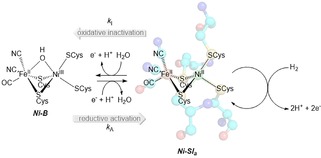
How the catalytically active Ni^2+^ form of the active site, the Ni‐Si_a_ state, can be reversibly inactivated to form the Ni^3+^‐containing Ni‐B state.

In O_2_‐sensitive [NiFe] hydrogenases such as *E. coli* Hyd‐2, the proximal cluster is a standard ferredoxin‐like [Fe_4_S_4_]^2+/1+^ center.^9^ However, crystal structures of O_2_‐sensitive [NiFe] hydrogenases also show a large‐subunit His residue close to the proximal cluster,2a,^9^ and amino acid sequence comparisons identify this highly conserved residue as HydC‐His214 in *E. coli* Hyd‐2 (Figure 1). Based on the Dance mechanism, it is postulated that removal of this His residue should not have a dramatic impact on enzymatic reactivity, because Hyd‐2 does not contain a [Fe_4_S_3_Cys_2_] proximal cluster. We test this theory by comparing the electrochemical activity of native Hyd‐2 enzyme and a Hyd‐2 HydC‐His214Ala variant (Hyd2‐H214A).

The procedure for the successful generation of variants in which His‐229 was replaced with Ala in *E. coli* Hyd‐1 and His‐214 was replaced with Ala in *E. coli* Hyd‐2 is described in SI. The purification of these variants and the “native” His‐containing unmodified versions of the enzymes was enabled by the incorporation of small subunit, C‐terminus hexa‐His tags (Figure S1). The purity of the isolated hydrogenases was checked with SDS‐PAGE gels, as shown in Figure S2. All purified enzymes contained a band at ∼65 kDa and a band at ∼40 kDa, denoting the large and small subunits, respectively, of the hydrogenase dimer. Additional protein bands were also visible, particularly in the Hyd‐1 variant and both Hyd‐2 samples. The band at ∼25 kDa has been identified in other hydrogenase samples and has been characterized as cAMP Receptor Protein, which has been named as one of the most common “contaminant” *E. coli* proteins seen in Ni‐affinity chromatography.^10^ The identity of all other bands is unknown although it is speculated that the additional band below the small subunit in the Hyd‐2 lanes is due to a degradation product formed during the O_2_‐exposed dialysis or centrifugation steps. In electrochemical experiments, the only detectable redox activity is assignable to hydrogenase catalysis indicating that either the contaminating proteins are non‐redox active or that they fail to adsorb to the electrode, with film formation essentially acting as a final purification step.

It is notable that the final protein yields of the variants were approximately half of those of the respective native enzymes (18 L bacterial growths yielded 0.6 mg native Hyd‐1, 0.3 mg variant Hyd1‐H229A, 6.8 mg native Hyd‐2 and 2.7 mg variant Hyd2‐H214A). It is therefore suggested that whatever the role in tuning chemical reactivity may be, in [NiFe] hydrogenases the structural integrity of the dimeric enzyme unit benefits from the presence of a large subunit His located at the large‐small protein dimer interface. Lower protein yields for a His‐to‐Ala variant were not noted in the purification of the *S. enterica* Hyd‐5,4c but this strain also contained an overexpression promoter at the start of the hydrogenase operon which might have masked this characteristic. Histidine has previously been noted as an important residue for protein stability, particularly as it may form multiple hydrogen bonds and exist in two tautomeric states.^11^

Figure 3 shows chronoamperometry experiments designed to monitor the impact of the His‐to‐Ala amino acid exchange on the sensitivity of the variant enzymes to O_2_. At pH 6.0 both native Hyd‐2 and the Hyd2‐H214A variant exhibit the characteristic aerobic reactivity of O_2_‐sensitive hydrogenases;^3^ upon addition of 3 % O_2_ into the gas mixture their catalytic activity tends towards zero (the native enzyme reaches 0 mA while the variant is at 4 % of the initial oxidation activity after 5 min exposure to a 3 % O_2_, 3 % H_2_ gas mixture). When O_2_ is removed from the gas stream, there is no significant difference in the extent of reactivation of native Hyd‐2 and Hyd2‐H214A, suggesting that the His does not act in a protective manner against formation of the slow‐reactivating, O_2_‐inihibited Ni−A state which O_2_‐sensitive [NiFe] hydrogenases are known to form.2a,^3^,^9^

As summarized in Table 1 and consistent with our work on *S. enterica* Hyd‐5, exchanging the conserved His for Ala in *E. coli* Hyd‐1 decreases O_2_ tolerance at pH 6.0.4c This was modelled by Dance as arising because deletion of the His impedes formation of the +5 oxidation state of the proximal cluster.^6^ The fact that the His to Ala mutation has a far more substantial impact on the O_2_ sensitivity of Hyd‐1 than Hyd‐2 at pH 6.0 supports this model. The impact of pH on the O_2_‐tolerance of Hyd‐1 is discussed later.

**Table 1:** Summary of chronoamperometry experiments quantifying the extent of O_2_ inhibition in native Hyd‐1 and the Hyd1‐H229A variant.

		Proportion of initial H_2_‐oxidation current sustained after 5 min 3 % O_2_, 3 % H_2_ [%]	Maximum proportion of recovered activity [%]
pH 4.5	Native Hyd‐1	30	93
	Hyd1‐H229A	21	94
pH 6.0	Native Hyd‐1	37	96
	Hyd1‐H229A	27	88
pH 7.6	Native Hyd‐1	40	90
	Hyd1‐H229A	24	88

Figure 2 shows that there is no significant difference in the catalytic wave shapes of native Hyd‐2 and the variant Hyd2‐H214A when the pH is changed from pH 6.0 to pH 7.6 in 3 % H_2_. Chronoamperometry experiments conducted at pH 4.5 (Figure S3) further confirm that the amino acid exchange has not impacted the catalytic bias, as do cyclic voltammograms under different partial pressures of H_2_ (Figure S4). Analogous catalytic bias potential‐step and voltammetric experiments comparing native Hyd‐1 and the Hyd1‐H229A variant also indicate that the His residue close to the proximal cluster plays a minimal role in controlling the ratio of oxidation to reduction current in Hyd‐1 (Figure 2, Figure S3 and Figure S4). Unlike our *Salmonella* Hyd‐5 experiments, we see no impact of the *E. coli* mutation on the apparent onset potential of catalysis in the O_2_‐tolerant MBH.4c

**Figure 2:**
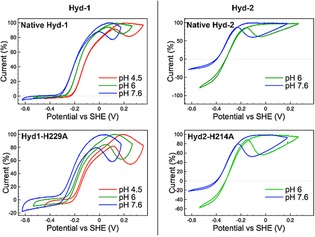
Catalytic voltammograms (5 mV s^−1^) measured under an atmosphere of 3 % H_2_, 97 % N_2_ for native Hyd‐1 (top left), Hyd1‐H229A variant (bottom left), native Hyd‐2 (top right), and Hyd2‐H214A variant (bottom right). Within each panel, the same film of enzyme was used for all the pH points. Four scans were measured at each pH and the fourth cycle is shown, with the current traces normalized to the maximum H_2_ oxidation current.

**Figure 3:**
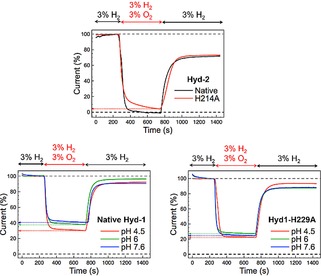
Chronoamperometric traces showing the O_2_ tolerance of native Hyd‐2 (black) and the Hyd2‐H214A variant (red) at pH 6.0 (top), as well as native Hyd‐1 (bottom left) and the Hyd1‐H229A variant (bottom right) at pH 4.5, 6.0 and 7.6, as indicated. For the Hyd‐2 experiments, the H_2_ oxidation activity was monitored at a constant potential of −0.135 V vs SHE; for the Hyd‐1 experiments, the potentials were +0.175 V vs SHE (pH 4.5), +0.085 V vs SHE (pH 6.0) or −0.05 V vs SHE (pH 7.6). Current traces were corrected for film loss and normalized to the H_2_ oxidation current immediately before the addition of O_2_.

At high potential there are distinct differences in the voltammogram shapes of native Hyd‐1 and variant Hyd1‐H229A across the pH range 4.5 to 7.6 (Figure 2). In all cases, the extent by which the current decreases at high potential, attributed to oxidative formation of the inactive Ni‐B state of the active site (Scheme 1), is more pronounced for the variant enzyme. This correlates with our earlier *Salmonella* Hyd‐5 work that compared the native enzyme to a His‐to‐Ala variant.4c Close examination of the work by Evans et al8b,^12^ also shows that greater inactivation is seen at high potentials in Hyd‐1 C19G/C120G and C19/C120G/P242C variants where the 4Fe3S proximal cluster could not form the superoxidized state. We interpret these facts as evidence that in O_2_‐tolerant MBH reversible interconversion between the catalytically active form of the enzyme and the oxidatively inactivated Ni‐B state involves formation of the +5 state of the proximal [Fe_4_S_3_Cys_2_] cluster even in the absence of O_2_. This is further supported by the fact that the high potential regions of the native Hyd‐2 and Hyd2‐H214A voltammograms are very similar, showing that the His residue close to the proximal cluster does not play a significant role in controlling the degree of Ni−B formation if the proximal electron‐transfer relay center is a [Fe_4_S_4_] cluster.

To further probe how the proximal cluster redox chemistry controls formation of the Ni‐B state, more extensive experiments were conducted on native Hyd‐1 and Hyd1‐H229A. In hydrogenase voltammetry, the parameter *E*_switch_ defines the potential of fastest reactivation in a scan from positive to negative potential after a prolonged oxidative potential‐poise inactivation period.^13^ A study by Fourmond et al^13^ concluded that this parameter is a feature of scan rate and the activation rate of the Ni‐B state, *k*_A_, where a 10‐fold increase in *k*_A_ shifts the *E*_switch_ by +80 mV.^13^ As shown in Figure S5, a potential poise of 5 hours inactivates the Hyd1‐H229A variant to a greater extent than native Hyd‐1, as would be expected based on the data in Figure 2. Extracting *E*_switch_ from the first derivative of these voltammograms reveals that at pH 4.5 and pH 6.0 the Hyd1‐H229A variant reactivates with *E*_switch_ values that are approximately 50 mV lower than the values for native Hyd‐1, which corresponds to a decrease in *k*_A_.^13^

Figure 4 shows the potential‐step experiment design and current‐time responses employed to extract anaerobic inactivation and reactivation rates, denoted *k*_I_ and *k*_A_, respectively. The method is based on that of Fourmond *et al*.^13^ The chronoamperometric traces for both native Hyd‐1 and variant Hyd1‐H229A at pH 6.0 and 10 % H_2_ were fit to the equations of Fourmond,^13^ as detailed in the Supporting Information (Figure S6), in order to derive the inactivation, *k*_I_, and reactivation, *k*_A_, rate constants as a function of potential (Scheme 1). The average rate constant values obtained from analyzing repeat experiments are shown in Figure 5. There is no consistent difference between the *k*_I_ of native Hyd‐1 and the Hyd1‐H229A variant that would explain the propensity of the variant to inactivate to a greater extent in cyclic voltammetry experiments. (Due to the contributions of background capacitive charging current to the data, we do not attach any significance to the small differences in *k*_I_, Figure S7.) Conversely, the value of *k*_A_ for the Hyd1‐H229A variant is consistently lower than that of native Hyd‐1. This correlates with the difference in *E*_switch_ values.

**Figure 4:**
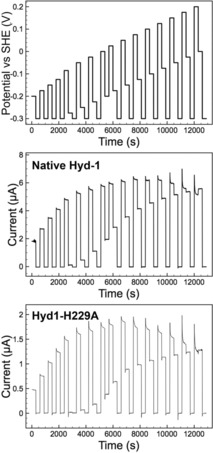
Potential step experiment to measure activation and inactivation rates of native Hyd‐1 and variant H1‐H229A at pH 6.0, 10 % H_2_. The potential‐time steps applied in the chronoamperometry experiment (top). Resulting chronoamperometric traces for native Hyd‐1 (middle) and variant Hyd1‐H229A (bottom). Other experimental conditions: 37 °C, electrode rotation rate 4,000 rpm, N_2_ carrier gas and total gas flow rate 1,000 scc min^−1^.

**Figure 5:**
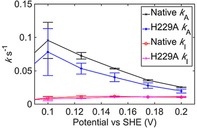
Inactivation, *k*_I_, and activation, *k*_A_, rate constant values for native Hyd‐1 and variant Hyd1‐H229A, as extracted from analysis of pH 6.0, 10 % H_2_ experiments such as those shown in Figure S6. Data points show the average value calculated from three repeat experiments, and error bars show the standard error.

According to the Dance model, removal of the His would impede the oxidative opening of the proximal cluster. Disrupting this process may decrease *k*_A_ due to the one electron Ni^3+/2+^ reductive activation of the active site proceeding via electron transfer from the proximal cluster oxidizing from the +4 to the +5 state. Alternatively, if the active form of the enzyme is assumed to require the proximal cluster in the +3 or +4 state, then slow reductive activation of the cluster may be interpreted as the reason for the variants decreased *k*_A_. It is suggested that to deconvolute the mechanism more precisely a more extended family of variants and wider suite of techniques is required. The amino acid residues which Dance predicts act as a communication relay between the active site and His‐229, particularly Thr‐80, which is thought to H‐bond to His‐229, should be exchanged for Ala or possibly valine in the presence and absence of the His‐to‐Ala exchange. Electrochemistry, EPR and ideally X‐ray crystallography data is then required to underpin more extensive calculations. Ideally, we would like to use the more sophisticated technique of Fourier transform large amplitude alternating current voltammetry to interrogate such variants, since this may enable measurement of the iron sulfur cluster redox transitions.2b,^14^

The fact that the inactivation rate *k*_I_ is insensitive to changes in the proximal cluster redox chemistry suggests that in the native and variant enzyme the rate‐determining step of oxidative inactivation remains unchanged. This can be interpreted by considering the work by Jones *et al*.^13^ They reported that in an O_2_‐sensitive [NiFe]‐hydrogenase the chemical binding of hydroxide was the rate‐limiting process during anaerobic oxidative inactivation, rather than the secondary electrochemical step.^13^

It is notable that in our work the impact of replacing His‐229 with Ala is most minimized at pH 4.5 in the Hyd‐1 O_2_ tolerance experiments (Table 1), whereas the native and variant enzymes have the most similar *E*_switch_ values at pH 7.5. Given the difference in experimental protocols and the fact that formation of the Ni‐B state will proceed via different mechanisms depending on the presence/absence of O_2_ we do not explain this observation in this communication. It suggests that extensive measurements of oxygenic inactivation rates at different potentials and different pHs should be carried out on the variant and native Hyd‐1.

Activation of the Ni‐B state would be presumed to proceed via the same mechanism under oxygenic or anaerobic conditions. Relating a slower rate of Ni‐B activation to increased O_2_‐sensitivity in a [NiFe] hydrogenase variant concurs with the original model of [NiFe]‐MBH O_2_‐tolerance proposed by Cracknell *et al*.^15^ Via comparison between native O_2_‐sensitive and O_2_‐tolerant enzymes it was concluded that a fast rate of Ni‐B reactivation is important in O_2_‐tolerance. In support of this, Hamdan *et al* 16 showed that in a series of hydrogenase variants, the ones with a higher Ni‐B reactivation rate had greater O_2_ tolerance. The mechanistic model is that rapid Ni‐B reactivation reflects the ability of the hydrogenase to achieve facile reduction of reactive oxygen species, and thus protection against O_2_. We note that this model and these results are contrary to a recent publication by Radu *et al*, in which the increased O_2_‐sensitivity of a [Fe_4_S_4_] proximal cluster variant of *R. eutropha* MBH was correlated with more rapid reactivation relative to the native, O_2_‐tolerant, [Fe_4_S_3_Cys_2_]‐containing enzyme.^17^ We cannot explain this, but emphasize that the *R. eutropha* work was uniquely conducted using a heterotrimeric hydrogenase complex in an artificial membrane with ubiquinone mediated electron transfer.^17^ In contrast, all other hydrogenase electrochemistry work (including our own) uses dimeric enzyme.

To conclude, our *E. coli* Hyd‐2 experiments indicate that in the presence of a standard [Fe_4_S_4_] proximal cluster, replacing the His near the proximal cluster with Ala has no major impact on catalysis or anaerobic or aerobic inactivation. Instead, the key role of this highly conserved His in O_2_‐sensitive enzymes appears to be the stabilization of the protein structure. Using *E. coli* Hyd‐1 and the Hyd1‐H229A variant we have addressed an oversight in previous work, where the role of the His in high potential anaerobic inactivation and reactivation was not quantified.4c,^5^, ^6^ Our new work explains that the reason the cyclic voltammogram shape of the *S. enterica* Hyd‐5 H229A variant changes relative to the native enzyme at high potentials4c is due to the impact of the amino acid exchange on the rate of reactivation of the Ni‐B state. Thus, the model by Dance, which considered how oxygenic inactivation of the NiFe active site could be communicated to the proximal cluster,^6^ is suggested to be relevant to reductive reaction in anoxygenic reactions. This indicates that future computational work probing the precise communication mechanism between the active site and the proximal cluster should not just consider how O_2_ binding at the active site triggers proximal cluster oxidation, but also how a hydroxide bound Ni^3+^ state is activated under anaerobic conditions.

## Conflict of interest

The authors declare no conflict of interest.

## Supporting information

As a service to our authors and readers, this journal provides supporting information supplied by the authors. Such materials are peer reviewed and may be re‐organized for online delivery, but are not copy‐edited or typeset. Technical support issues arising from supporting information (other than missing files) should be addressed to the authors.

Supplementary
