# Supplementary material for: Conserved Histidine Adjacent to the Proximal Cluster Tunes the Anaerobic Reductive Activation of Escherichia coli Membrane‐Bound [NiFe] Hydrogenase‐1
Source: ChemElectroChem. 2018 Feb 16;5(6):855–60. doi: 10.1002/celc.201800047 (PMC5900901; doi:10.1002/celc.201800047)
Supplement: Supplementary file 1 — Supplementary [file CELC-5-855-s001.pdf]

Supporting Information

Contents

Experimental Section 2
- Molecular biology 2
- Protein purification 3
- Protein film electrochemistry 4

Protein gel 6

Experiments probing the impact of the histidine to alanine substitution on catalytic bias 7

Comparing E_{switch} of native Hyd-1 and variant Hyd1-H229A 9

Extracting anaerobic inactivation and reaction rate constants, k_I and k_A, from potential step experiments 10

Reference electrode calibration 13

References 14

Experimental Section

Molecular biology

Biosynthesis of an intact MBH requires the expression of at least two separate gene operons. The first operon encodes the subunits of the hydrogenase of interest and several accessory proteins which act as endopeptidases and chaperones.[1] The second operon is the Hyp operon, which encodes the proteins that assemble the complex structure of the hydrogenase active site.[1] Further to this are the proteins, not yet fully understood, that perform the synthesis and insertion of the iron-sulfur clusters.[1] Because this maturation process is so complex, chromosomal mutations are made to *E. coli* Hyd-1 and Hyd-2. The Heerman methodology[2] has therefore been used to add polyhistidine tags and create single site variants.

The *E. coli* strains used in this study are LAF-003, LAF-018, LAF-019 and LAF-023. The parent "LAF-001" strain is an *E. coli* K12 strain W3110[3] which has been modified to contain the *rpsL*150 allele from MC1061[4] using Red®/ET® recombination, as described previously.[5]

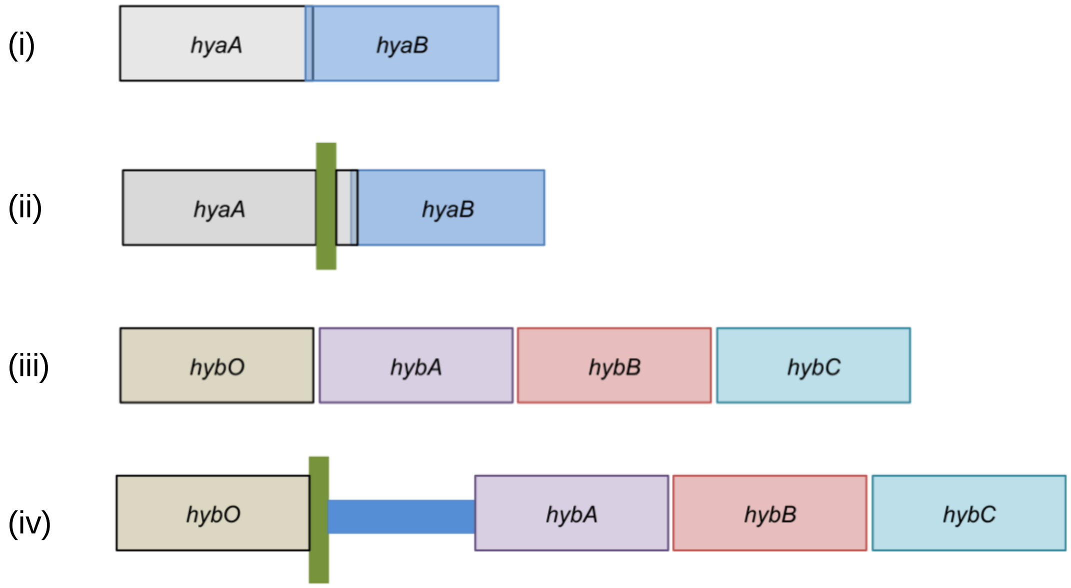

Figure S1 Schematic depiction of the insertion of a polyhistidine tag into the gene operons of Hyd-1 and Hyd-2. Vertical green bar represents the nucleotides coding for the polyhistidine tag plus stop codon whilst horizontal blue bar represents the *rpsL-neo* cassette. (i) wild-type small and large subunit Hyd-1 genes, *hyaA* and *hyaB*. Note the overlap of the stop and start codons; (ii) insertion of nucleotides coding for polyhistidine tag at the C-terminus of the small subunit of Hyd-1, and 20 base pairs duplicated from the terminus of *hyaA*; (iii) wild type small and large subunit Hyd-2 genes, *hybO* and *hybC*. Note the separation of these genes; (iv) insertion of nucleotides coding for polyhistidine tag at the C-terminus of the small subunit of Hyd-2, and cassette.

Creation of LAF-003, which expresses “native” His-tagged Hyd-1 has been detailed before.[5] Strain LAF-018 is derived to express the HyaB-His229Ala His-tagged Hyd-1 variant (referred to as “H229A”), in a similar manner to which other Hyd-1 point mutation variants have been created.[5] Because the stop codon for the gene for the small subunit, *hyaA*, overlaps with the start codon for the large subunit of Hyd-1, *hyaB* the codons for the polyhistidine tag are located after the bases coding for the C-terminus of *hyaA*, and the final 20 bases of *hyaA* are duplicated after in both strains (Figure S1).

Strain LAF-019, which expresses the His-tagged “native” Hyd-2, and strain LAF-023, which expresses the HybC-His214Ala His-tagged Hyd-2 variant, have not previously been described. The synthesis of these took advantage of the fact that in the *hyb* operon of *E. coli* the *hybC* gene encoding the large subunit and the *hybO* gene encoding the small subunit of the hydrogenase are separated by genes *hybA* and *hybB* (Figure S1).[6] Native Hyd-2 and the variant H2-H214A proteins could therefore both be purified from strains where the chromosome contained nucleotides coding for the polyhistidine tag immediately followed by the *rpsL-neo* cassette (Figure S1). DNA sequencing (data not shown) was used in all cases to verify the hydrogenase genetic changes.

Protein purification

The protein purification protocol was adapted from Lukey et al.[7] 10 mL starter cultures of the native and variant Hyd-1 strains were grown in Luria broth (LB) containing streptomycin (50 μg mL^{-1}) at 37 °C with shaking for 6 hours. 3 mL of this starter culture was then used to inoculate 6 L bottles containing LB supplemented with glycerol (1% v/v), sodium fumarate (0.4% w/v) and streptomycin (50 μg mL^{-1}). The bottles were filled to leave only 5 mL headspace and placed in a standing incubator at 37 °C overnight until an optical density (OD) of ~1 was reached. The Hyd-2 strains were grown in streptomycin-free media using the same procedure.

Following growth, the cells were harvested by centrifugation (5,500x*g* for 15 min, 4 °C), and the pellet was resuspended in chilled buffer (100 mM Tris pH 7.6, 300 mM NaCl). Sucrose (20% w/v) was added to the resuspension and this mixture was stirred at 4 °C for at least 45 min. Following centrifugation (6500x*g* for 20 min at 4 °C) cells were lysed by osmotic shock via resuspension in 300 mL ice-cold water and stirring at 4 °C for at least 30 min. Solubilisation of the membranes was then achieved by adjusting the solution to 100 mM Tris pH 7.6, 300 mM

NaCl, 9% (v/v) Triton X-100 and finally adding lysozyme (5 mg, Sigma Aldrich), Deoxyribonuclease I (Sigma Aldrich) and Pierce™ Protease Inhibitor Mini Tablets, EDTA-free (Thermo Scientific) to the cell suspension before stirring for 16 h at 4 °C. To ensure total cell lysis a final sonication step was performed (20 mm probe using a Soniprep 150 (MSE) at a power of 15-20 µm on ice for 10 x 30 s) followed by centrifugation (20000x*g*, 30 min at 4 °C) to obtain soluble extract.

Soluble cell extract was prepared for chromatography by diluting to a final concentration of 150 mM NaCl (by addition of 100 mM Tris pH 7.6) and the addition of imidazole to 50 mM. The resultant solution was then loaded onto a 5mL HisTrap Ni affinity column (GE Healthcare) equilibrated in loading buffer (100 mM Tris, 150 mM NaCl, 50 mM imidazole, pH 7.6) at 4 mL min^{-1} (AKTA Start, GE Healthcare). Following washing in loading buffer, protein was eluted using a 0-100% linear gradient in elution buffer (100 mM Tris, 1 M imidazole, pH 7.6, 50 mL). The presence of protein was detected by UV absorbance at 280nm. Column fractions containing hydrogenase were identified by SDS PAGE, and dialyzed overnight at 4 °C (100 mM Tris, 150 mM NaCl, pH 7.6). Purified protein was concentrated (30kDa Amicon Ultra centrifugal filter, Merck Millipore) to 0.1-0.3 mgmL-1 (determined by PierceTM Coomassie Protein assay Kit, Thermofisher) and stored at -80 °C.

Protein film electrochemistry

Protein film electrochemistry was performed in a N_2-filled glove box (Faircrest). A gas tight electrochemical cell (built in-house by the University of York glassblowers) housed the three electrode configuration. The saturated calomel reference electrode (SCE) was held in a side arm filled with 0.1 M NaCl and connected to the main body of the cell by a Luggin capillary. A water jacket enabled heating of the working and counter electrode compartment to 37 °C. All experiments were conducting using a "mixed hydrogenase" buffer in this compartment of the electrochemical cell. This buffer comprised 15 mM sodium acetate (Sigma Aldrich), CHES (N-Cyclohexyl-2-aminoethanesulfonic acid) (AMRESCO), MES (2-(N-morpholino)ethanesulfonic acid) (Sigma Aldrich), HEPES (4-(2-hydroxyethyl)-1-piperazineethanesulfonic acid) (Sigma Aldrich) and TAPS (N-tris(Hydroxymethyl)methyl-3-propanesulfonic acid sodium potassium salt) (Sigma Aldrich), and 100 mM NaCl. The pH of the buffer was adjusted using HCl and NaOH, and approximately 2.5 mL was used to cover the electrodes in the main body of the cell. The counter electrode was a piece of platinum wire and the graphite working electrode were manufactured in-house at the University of York. Gases (BOC) were flowed through the

electrochemical cell at a total gas flow rate of 100 scc min^{-1} under the control of Smart-Trak mass flow controllers (Sierra Installations) connected to the electrochemical cell. N_2 was used as a carrier gas.

To prepare the graphite working electrode for hydrogenase adsorption, Norton P1200 abrasive sheets were used to sand the surface before application of 2 μL of enzyme. A CompactStat potentiostat (Ivium Technologies) and the IviumSoft Program were used to control the electrochemical experiment and unless stated otherwise the electrode was rotated at 4,000 rev min^{-1} using an Origatrod rotator (Origalys) to allow an adequate supply of substrate and removal of product. All cyclic voltammetry was performed at 5 mV s^{-1} unless specified otherwise.

A reference electrode correction factor was determined via calibration measurements made using 100 μM methylene blue cyclic voltammetry at pH 7, 25 °C with a platinum working electrode (Figure S8). This yielded the following correction, which has been used to adjust all measured potentials to V vs SHE: E(V vs SHE) = E(V vs Ref) +0.265 V.

Protein gel

It is likely that the additional band below the small subunit for the Hyd-2 samples is due to a degradation product formed during the O_2-exposed dialysis or centrifugation steps. The band at ~25 kDa has been identified in other hydrogenase samples and has been characterised as cAMP Receptor Protein, which has been named as one of the most common "contaminant" proteins seen in Ni-affinity chromatography.[8]

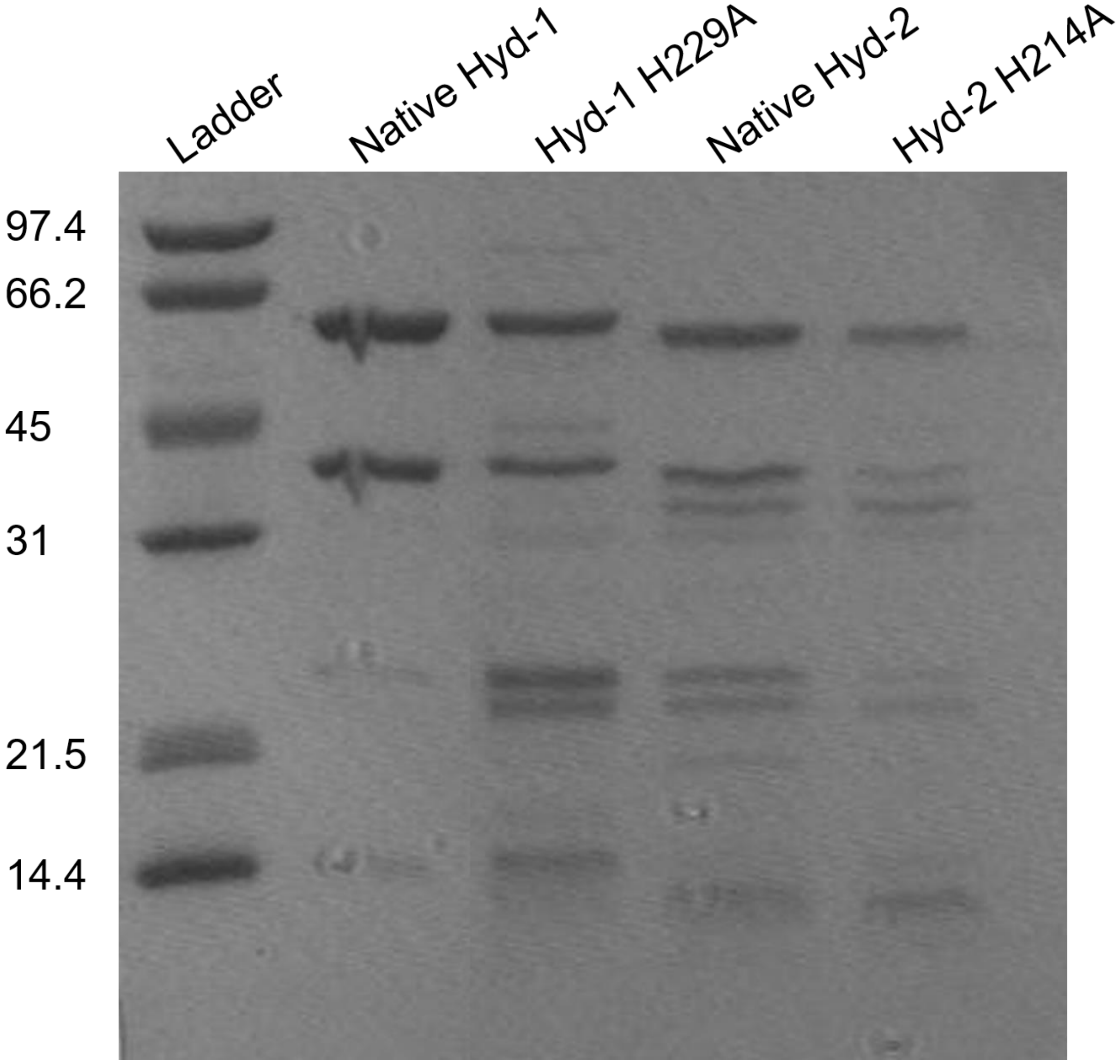

Figure S2 SDS PAGE gel loaded with concentrated Hyd-1 and Hyd-2 native and His to Ala variant hydrogenase, as indicated. The strong band at 65 kDa corresponds to the large subunit whilst the strong band at 40 kDa corresponds to the small subunit.

Experiments probing the impact of the histidine to alanine substitution on catalytic bias

Figure S3 shows chronoamperometry experiments in which the potential is switched from reducing to oxidizing and back again at 0% H_2, then to oxidizing potential followed by reducing potential at 3% H_2. The traces were normalized to the current of maximum H_2 oxidation. Such experiments confirm that the histidine to alanine amino acid exchange has a minimal impact on catalytic bias for both Hyd-1 and Hyd-2. Both Native Hyd-2 and the Hyd2-H214A variant are bidirectional hydrogenases,[7] with the large negative current indicating substantial H_2 production at low potential under both 0 and 3% H_2. When the rotation of the working electrode is ceased (red bar), H_2 production by Hyd-2 is less product inhibited than Hyd-1, something which has been described previously.[7]

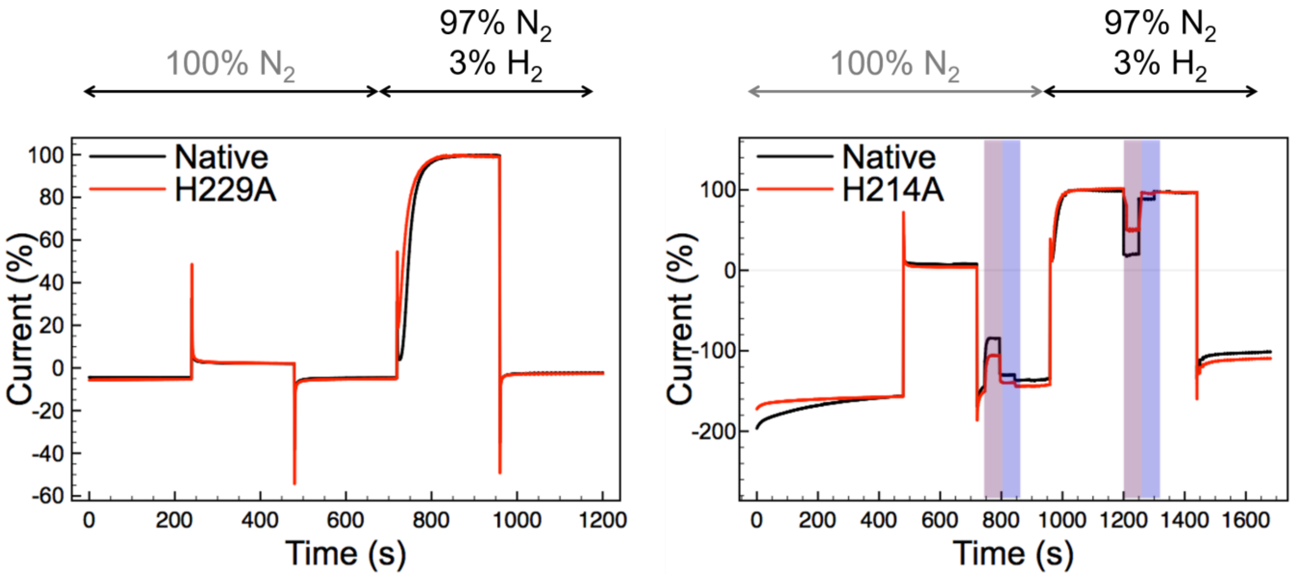

Figure S3 Chronoamperometric trace overlay of H_2 production in native (black) and His to Ala variant (red) of (left) Hyd-2 and (right) Hyd-1. All experiments were conducted at pH 4.5 and 37 °C. The potential was switched between reducing and oxidizing potentials and the gas in the cell was switched from 100% N_2 to 97% N_2 and 3% H_2 (total gas flow rate maintained at 1,000 scc min^{-1}). The current trace is normalized to the maximum H_2 oxidation activity under 3% H_2. Negative current indicates H_2 production. For the Hyd-2 experiment, the potential was switched between -0.4 V vs SHE and -0.045 V vs SHE. Colored vertical bars show where the electrode rotation was stepped from 4000 rev per min to 0 rpm (purple) and then to 1,000 rpm (blue). For the Hyd-1 experiment the potential was switched between -0.4 V vs SHE and +0.175 V vs SHE and the electrode rotation was maintained at 4,000 rpm throughout.

Figure S4 shows cyclic voltammetry experiments conducted under different partial presses of H_2 which further contrast the bidirectional catalytic activity of Native Hyd-2 and the

Hyd2-H214A variant with the unidirectional oxidation-only catalysis of Hyd-1 and the Hyd1-H229A variant.

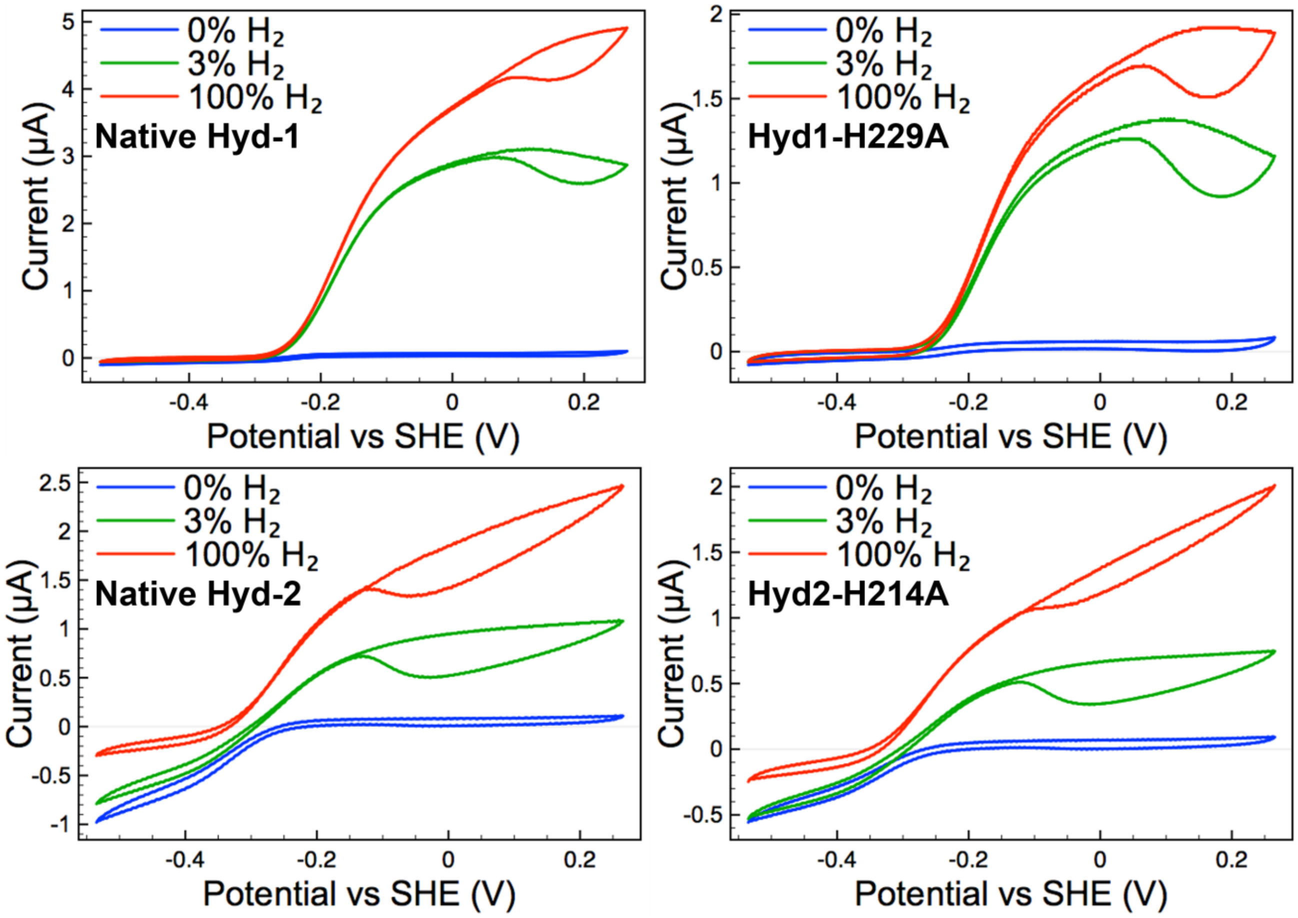

Figure S4 Catalysis of native Hyd-1 and Hyd-2 and their respective His-to-Ala variants under different partial pressures of H_2. The potential was swept from -0.445 V vs SHE to +0.355 V vs SHE at 5 mV s^{-1} before being swept back to the low potential. This scan was repeated four times for each percentage of H_2 and the fourth cycle is shown. Experiments conducted at pH 6.0, 37 °C, electrode rotation rate 4,000 rpm, N_2 carrier gas and total gas flow rate 1,000 scc min^{-1}.

Comparing E_{switch} of native Hyd-1 and variant Hyd1-H229A

Figure S5 shows voltammograms measured after an oxidative inactivation high-potential poise. These voltammograms are analyzed to extract the plotted E_{switch} values via determination of the potential at which there is a maxima in the first derivative of the reductive activation current.

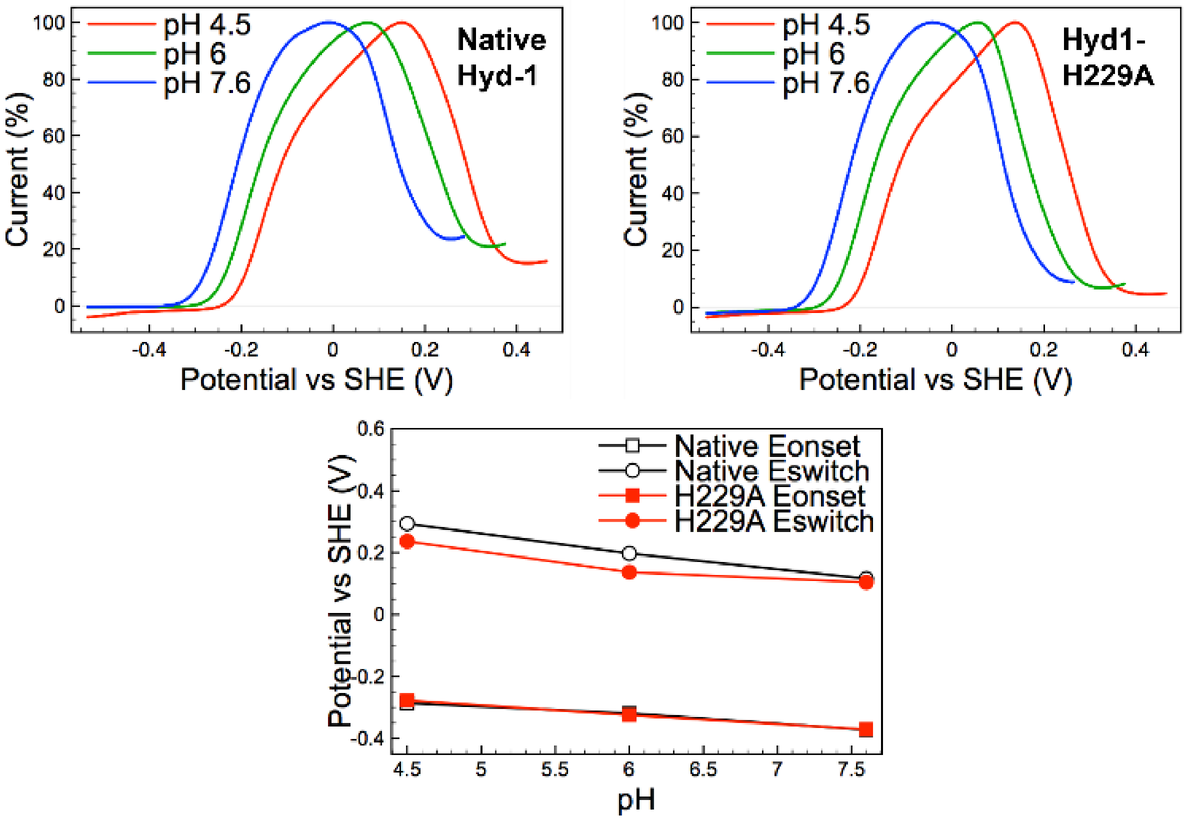

Figure S5 Determining E_{switch} of (top left) native Hyd-1 and (top right) variant Hyd1-H229A in 3% H_2 and different pH, as indicated. Following a 5-hour potential poise at either +0.465 V vs SHE (pH 4.5), +0.375 V vs SHE (pH 6.0) or +0.285 V vs SHE (pH 7.6) the potential was swept to -0.535 V vs SHE at 0.2 mV s^{-1}. Current is normalized to the maximum current. (Bottom) Comparison of the extracted E_{switch} values. Other experimental conditions: 37 °C, electrode rotation rate 4,000 rpm, N_2 carrier gas and total gas flow rate 1,000 scc min^{-1}.

Extracting anaerobic inactivation and reaction rate constants, k_I and k_A, from potential step experiments

Current-time data from the chronoamperometry experiments shown in Figure 4 was separated into the response from each individual potential step using MATLAB 2016a software (MathWorks). The timespan of each individual potential poise was established as a condition using the "cond" function, and the start point was assigned as 0 sec and end point 300 sec. Each inactivation dataset (current-time trace that resulted following a step to high potential from a potential of -0.3 V) was then fit to Equation 1[9] using the code listed, where xdata is the time in seconds and the ydata is the experimental current. Equation 1 is defined in the fittype function, where a is i_0, b is i_∞ and c is k_{tot}. A typical fit given by this method is shown in Figure S6, and it may be seen that the experimental data (blue) and simulated data ("fit", red) overlap closely.

$$i(t) = (i_0 - i_\infty)\exp[-k_{tot}E(t)] + i_\infty \qquad \text{Equation 1}$$

MATLAB code:

```
fo = fitoptions('Method','NonlinearLeastSquares',...
            'Lower',[0,0],...
            'Upper',[Inf,max(xdata)],...
            'StartPoint',[1 1 1]);
ft = fittype('((a-b)*exp(-c*x)+b )','options',fo);
 [curve2,gof2] = fit(xdata,ydata,ft);
c=coeffvalues(curve2);
t=table(43,c(1),c(2),c(3),'VariableNames',{'E' 'i0' 'iinf' 'ktot'});
```

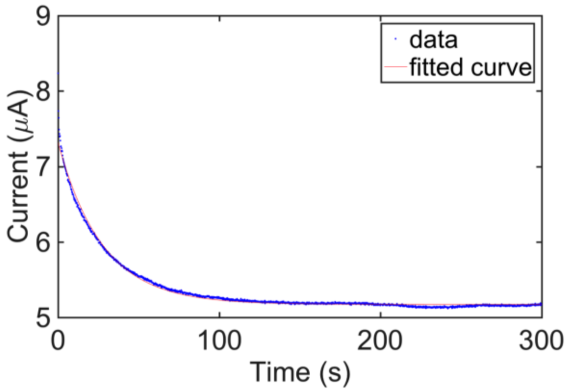

Figure S6 Fit versus data for the inactivation curve. The fit of Equation 5.1 (red line) against the current data (blue trace) for Native Hyd-1 when the potential is stepped to +0.2 V vs SHE from -0.3 V vs SHE and sustained at +0.2 V vs SHE for 300 seconds.

The MATLAB coeffvalues function allowed the coefficients a, b and c of the best fit for each inactivating potential step to be to be written to a table. The separate tables of coefficients for each repeat experiment were aggregated by compiling them into a MATLAB datastore and then writing the contents of the datastore as an Excel workbook (Microsoft). Using this data, the potential dependent inactivation rate (k_I) and reactivation rate (k_A) values were calculated using Equation 2 and Equation 3,[9] the MATLAB derived k_{tot} values and assuming $A_0 = 1$ (i.e. assuming that the enzyme is 100% active at the start of the poise at high potential because the inactivation step immediately followed a reductive activation potential poise).

$$k_A(E) = A_0 \frac{i_\infty}{i_0} k_{tot}(E) \qquad \text{Equation 2}$$

$$k_I(E) = k_{tot}(E) - k_A(E) \qquad \text{Equation 3}$$

Figure S7 shows data from a “control”, enzyme-free, potential-step experiment.

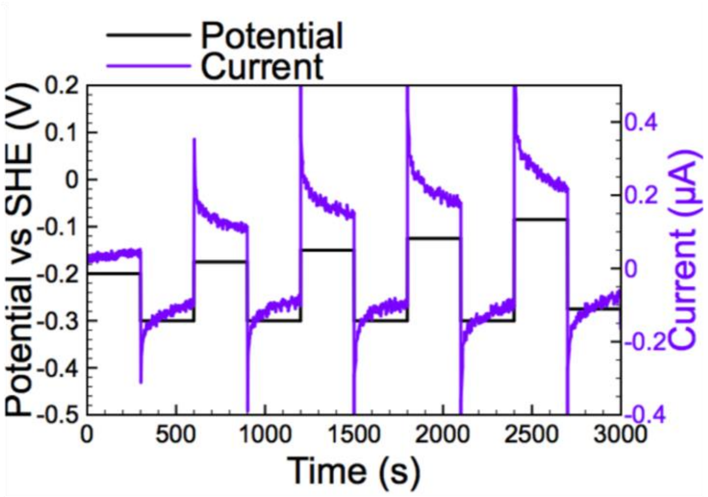

Figure S7 Chronoamperometric response of an enzyme-free "blank" electrode under the same conditions as the experiments in Figure 4. Black trace shows the steps in electrode potential, purple trace shows the resulting current.

Reference electrode calibration

A reference electrode correction factor was determined via calibration measurements made using 100 µM methylene blue cyclic voltammetry at pH 7, 25 °C with a platinum working electrode (Figure S8).

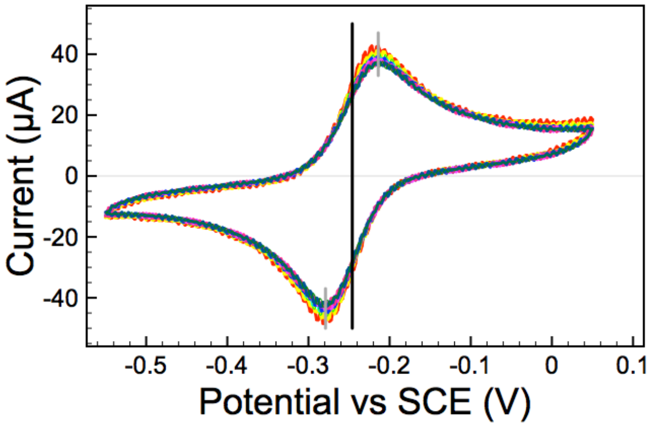

Figure S8 Calculation of the correction factor. The potential was swept from -0.56 to +0.06 V vs SCE and back again at 50 mV s^{-1} using the platinum wire as a working electrode. The potential of the maximum and minimum current are marked by grey lines and the midpoint potential of -0.246 V vs SCE is given by the black line.

The values given for the potentials of maximum current (E at imax), minimum current (E at imin), midpoint (Emid), and correction factor (Ecorrection) needed to give the value of Em,7 = +0.019 V vs SHE (calculated from published reference data[10]) are given in Table S1. The correction value used was E(V vs SHE) = E(V vs Ref) +0.265 V.

Table S1 Values used to determine the reference electrode correction factor.

E at imax	E at imin	Emid	Ecorrection
-0.214	-0.282	-0.248	0.267
-0.214	-0.276	-0.245	0.264
-0.212	-0.278	-0.245	0.264
-0.216	-0.280	-0.248	0.267
-0.214	-0.276	-0.245	0.264
-0.218	-0.280	-0.249	0.268
-0.214	-0.276	-0.245	0.264
-0.212	-0.278	-0.245	0.264
-0.208	-0.282	-0.245	0.264

References

[1] L. Bowman, J. Balbach, J. Walton, F. Sargent, A. Parkin, *J. Biol. Inorg. Chem.* **2016**, *21*, 865-873.

[2] R. Heermann, T. Zeppenfeld, K. Jung, *Microb. Cell Fact.* **2008**, *7*, 14.

[3] K. Hayashi, N. Morooka, Y. Yamamoto, K. Fujita, K. Isono, S. Choi, E. Ohtsubo, T. Baba, B. L. Wanner, H. Mori, T. Horiuchi, *Mol. Syst. Biol.* **2006**, *2*.

[4] M. J. Casadaban, S. N. Cohen, *J. Mol. Biol.* **1980**, *138*, 179-207.

[5] L. A. Flanagan, J. J. Wright, M. M. Roessler, J. W. Moir, A. Parkin, *Chem. Commun. (Cambridge, U. K.)* **2016**, *52*, 9133-9136.

[6] A. Dubini, R. L. Pye, R. L. Jack, T. Palmer, F. Sargent, *Int. J. Hydrogen Energy* **2002**, *27*, 1413-1420.

[7] M. J. Lukey, A. Parkin, M. M. Roessler, B. J. Murphy, J. Harmer, T. Palmer, F. Sargent, F. A. Armstrong, *J. Biol. Chem.* **2010**, *285*, 3928-3938.

[8] C. Robichon, J. Luo, T. B. Causey, J. S. Benner, J. C. Samuelson, *Appl. Environ. Microbiol.* **2011**, *77*, 4634-4646.

[9] V. Fourmond, P. Infossi, M.-T. Giudici-Orticoni, P. Bertrand, C. Léger, *J. Am. Chem. Soc.* **2010**, *132*, 4848-4857.

[10] A. Hulanicki, S. Głąb, *pac* **1978**, *50*, 463.
